# Residual tensile force estimation method for earth anchor using elasto-magnetic sensing system

**DOI:** 10.1371/journal.pone.0264078

**Published:** 2022-03-01

**Authors:** Sehwan Park, Junkyeong Kim, Changgil Lee

**Affiliations:** 1 Safety Inspection for Infrastructure Laboratory, Advanced Institute of Convergence Technology, Suwon-si, Gyeonggi-do, South Korea; 2 Advanced Railroad Civil Engineering Division, Korea Railroad Research Institute, Uiwang-si, Gyeonggi-do, South Korea; Fuzhou University, CHINA

## Abstract

The earth anchor method is used to prevent landslides, and repair and reinforce cut or steep slopes due to its benefits of ease of construction and economic feasibility. However, the loss of anchor force has become a problem, which may cause failure and collapse of slopes when the anchor force drops below the design anchor force. While numerous studies have been conducted to solve this problem, measuring the residual tensile force of existing earth anchors remains a challenge, as prior studies required sensors to be installed inside structural members at the time of construction. Therefore, to address this limitation, an experiment was performed in this study to develop an elasto-magnetic (EM) sensor for measuring tensile force based on the EM effect, which could be installed on externally exposed anchor heads. The commercial software ANSYS Maxwell was used to analyze the optimal sensor design for the experiment. Additionally, a series of tests to measure the tensile force was conducted by fabricating the sensor based on the numerical analysis results. The area of B-H curves measured by developed EM sensor was increased according to the decrease of tensile force. Also, The tensile force estimation equation was derived and verified using measured data. According to the results, the proposed method can be one of the solution for measuring residual tensile force of earth anchor.

## Introduction

More than 63% of the land area in South Korea consists of mountains with shallow ground surfaces that are highly vulnerable to collapse of steep slopes. These steep slopes, including cut slopes and retaining walls, have been rapidly increasing because of urbanization and industrialization. A slope with a vertical height of at least 30 m from the ground surface (or from the top of the retaining wall, if applicable) and a single horizontal length of 100 m or greater is classified as a cut slope.

As cut or steep slopes can collapse during the thawing season every year, safety inspections are undertaken in the corresponding periods followed, by repair and reinforcement measures based on the inspection results. However, collapse accidents continue to occur as the repair and reinforcement of cut or steep slopes with safety concerns are delayed due to financial reasons. Steep slope collapses that occurred in the past decade involved cut slopes (37 cases, 51.4%), retaining walls (14 cases, 19.4%), construction sites (13 cases, 18.1%), and others (8 cases, 11.1%).

The earth anchor (or ground anchor) method to prevent such collapses is often used for repairing and reinforcing cut or steep slopes owing to the advantages of ease of construction and economic feasibility. An anchor is a structural element fixed in the ground with good load bearing capacity at the fore-end to support structures, such as earth walls, using reaction forces. The anchor consists of an anchor body formed by grouting, a tensile part that transfers the anchor force of the anchor body, and an anchor head that fixes the tensile part to the structure [1].

However, the anchor force can be reduced due to some causes, such as relaxation of anchor tendons, slope surface irregularity caused by displacement near anchor heads, surface strength, friction loss due to displacement near anchorage, short-term creep, etc. [2, 3]. The resulting reduction in anchor force below the design anchor force will probably cause slopes to fail or collapse. Protrusions due to anchor failures, cracks and damages of pressure plates or pressure structures, and damages of anchor head caps or head concrete can be monitored to determine the current condition of anchors. However, it is still difficult to confirm the degradation in performance associated with the residual anchor force, which results in the collapse.

The conventional test methods for measuring residual anchor force include the performance test(or suitability test), pull-out test, lift-off test, and proof test(or acceptance test). The methods for measuring residual anchor force during the maintenance of anchors include the method of installing load cells while anchor tensile force is being introduced and the residual tensile force measurement method.

However, the conventional measurement of residual anchor force is impossible for anchors with no load cells installed during construction. Additionally, some anchors are not monitored and managed due to practical challenges, such as the need to replace load cells that are past their life span and the time required to measure the anchor force. Therefore, the residual anchor force decreases by 24–70% compared to the design anchor force. Moreover, some anchors appear to have a level of slope stability that is significantly below the standard safety limit in terms of residual anchor force [4].

Globally, research is underway to address these problems. Studies have been conducted on methods for designing a shaft-style jack anchor to reduce the loss of tensile force in earth anchors [5], measuring anchor loads via load tests using a fiber Bragg grating (FBG) sensor attached to the surface of existing load cells [6], performing pull-out tests to observe the anchor behavior by installing anchors of different types (tension, compression, and composite anchors) at different depths [7], and measuring anchor prestressed force using wireless force sensors [8] and FBG sensors [9, 10]. Domestic studies include the measurement of anchor axial force and changes in prestressed force using load cells on anchor heads [11, 12] and the measurement of load transfer distribution of tension and compression anchors using strain gauges, embedded gauges, load cells, etc. [13–15].

However, the measurement of residual tensile force of previously constructed anchors remains limited because prior studies mainly suggest the method of embedding magnetic sensors attached to tendons during the construction of earth anchors to measure tensile force.

**Fig 1:**
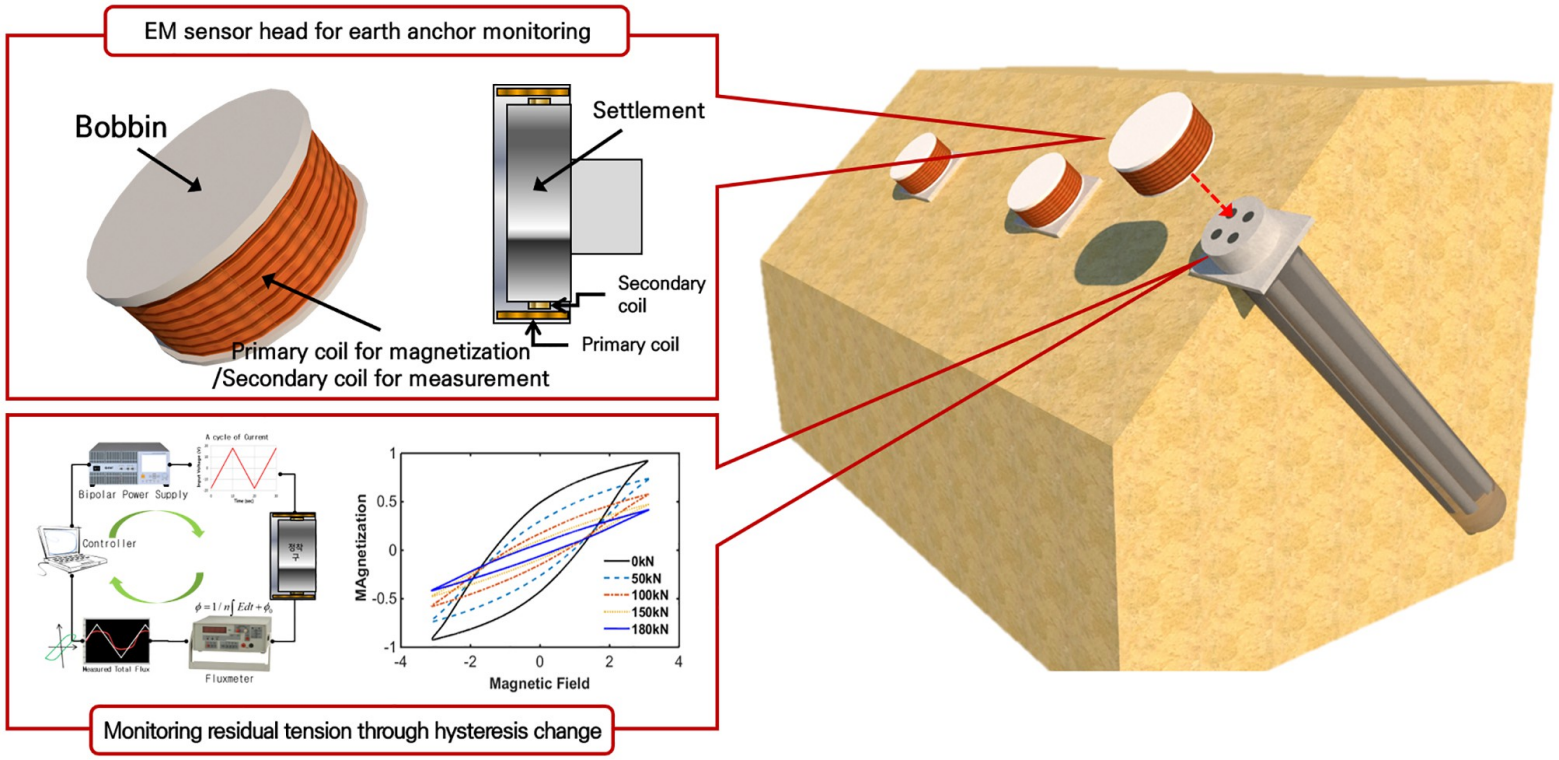
Schematic showing the residual anchor force monitoring system of the earth anchor using the magnetic-elastic effect.

To overcome the disadvantages and limitations of the existing technology, an experiment was performed in this study to develop an elasto-magnetic (EM) sensor for measuring tensile forces based on the EM effect [16]. This sensor can be installed on externally exposed anchor heads, as shown in Fig 1, rather than being directly attached sensor system to the earth anchor tendon and embedded together.

In this study, the variation in load with the tensile force of the earth anchor was first identified via simulation. The inner and outer diameters of the bobbin, input wavelength, and current were designed to magnetize the earth anchor head from the results of the numerical simulation. Additionally, based on the simulation results, a field experiment was conducted to verify the fabricated sensor using earth anchor specimen. This was followed by an experiment to diagnose the residual tensile force of the earth anchor by evaluating the change of magnetic hysteresis with the tensile force.

## Theoretical background about EM sensor

The length of a ferromagnetic material changes when placed in a magnetic field. This phenomenon is referred to as magnetostriction. The mechanical strain experienced during this process can be calculated using Eq (1).

$$\begin{array}{c} \lambda =\frac{\Delta l}{l} \end{array}$$ (1)

where *l* is the initial length of the ferromagnetic material, and Δ*l* is the strain due to the magnetization. A material that has positive magnetostrictive properties increases in length when magnetized. When a prestressed force is applied while the material is magnetized, it elongates the material and increases the magnetic flux of the material.

Ferromagnetic materials are magnetized when placed within an aligned magnetic field. The stronger the magnetic field is applied to the test specimen, more the magnetic domains are aligned and stronger the magnetization becomes, eventually reaching a point where all domains are aligned. After that, no magnetization occurs. This point is referred to as magnetic saturation [17].

**Fig 2:**
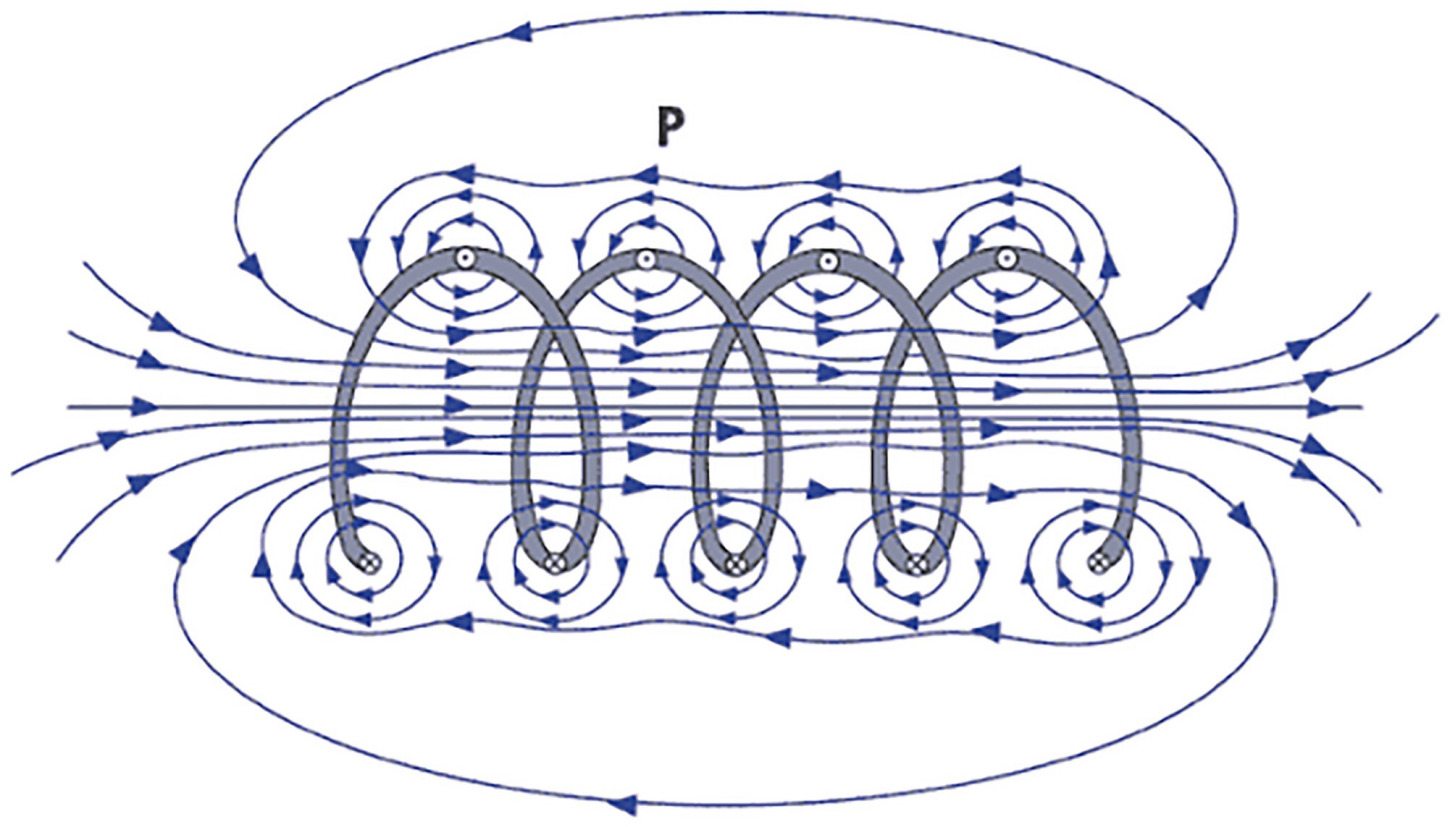
The magnetic field generated by the solenoid coil.

As shown in Fig 2, when the current flows into a solenoid coil, the concentrated magnetic field flows through the center of the coil. The magnetic field formed in the solenoid *H* is calculated as *H = NI*, where *N* is the number of turns per meter, and *I* is the magnitude of the current (*A*) applied to the solenoid.

Based on this principle, a magnetic field of arbitrary strength can be generated according to the test specimen and test conditions, where the strength of the magnetic field depends on the type of core, turn diameter, turn number, magnetic path (core shape), and input current magnitude [18, 19].

### Acquisition and properties of a magnetic hysteresis curve

A magnetization curve, also referred to as a magnetic hysteresis curve, shows the relationship between the magnetic field force and magnetic flux density of a ferromagnetic material. Additionally, it is used to indicate the magnetic properties of ferromagnetic material [20].

In this study, the hysteresis curve was evaluated to represent the magnetic properties of the earth anchor tendon, which was composed of a ferromagnetic material. The changes in magnetic properties of the tendon with respect to tensile force were obtained to apply the pattern of changes in this study.

**Fig 3:**
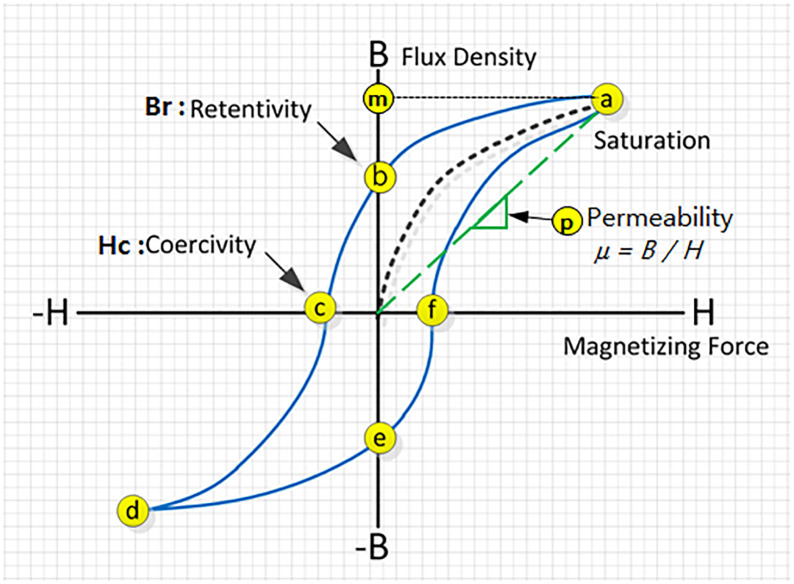
Hysteresis curve.

Generally, a magnetic curve, whose shape is shown in Fig 3, is acquired through the process described below. The subject was placed in the magnetic field and saturated by applying a current to the magnetization coil (the primary coil) to generate the magnetic field. To reverse the magnetic field of the subject, the magnetic flux direction was reversed by applying a reverse magnetic field to the primary electromagnetic coil.

The magnetic field strength required to reverse the flux direction of the subject is the intrinsic coercive force. In other words, the magnetic hysteresis curve represents the numerical values measured on the search coil (the secondary coil to measure the magnetic flux density applied to the subject) installed on the subject over a single cycle of saturating the magnetic field in the positive direction, i.e., from the electromagnet → gradually to zero → saturating the magnetic field in the negative direction → gradually to zero. The residual magnetic flux density, intrinsic coercive force, and permeability derived from the magnetic hysteresis curve can be used to detect the changes in the cross-section and material properties [21].

**Fig 4:**
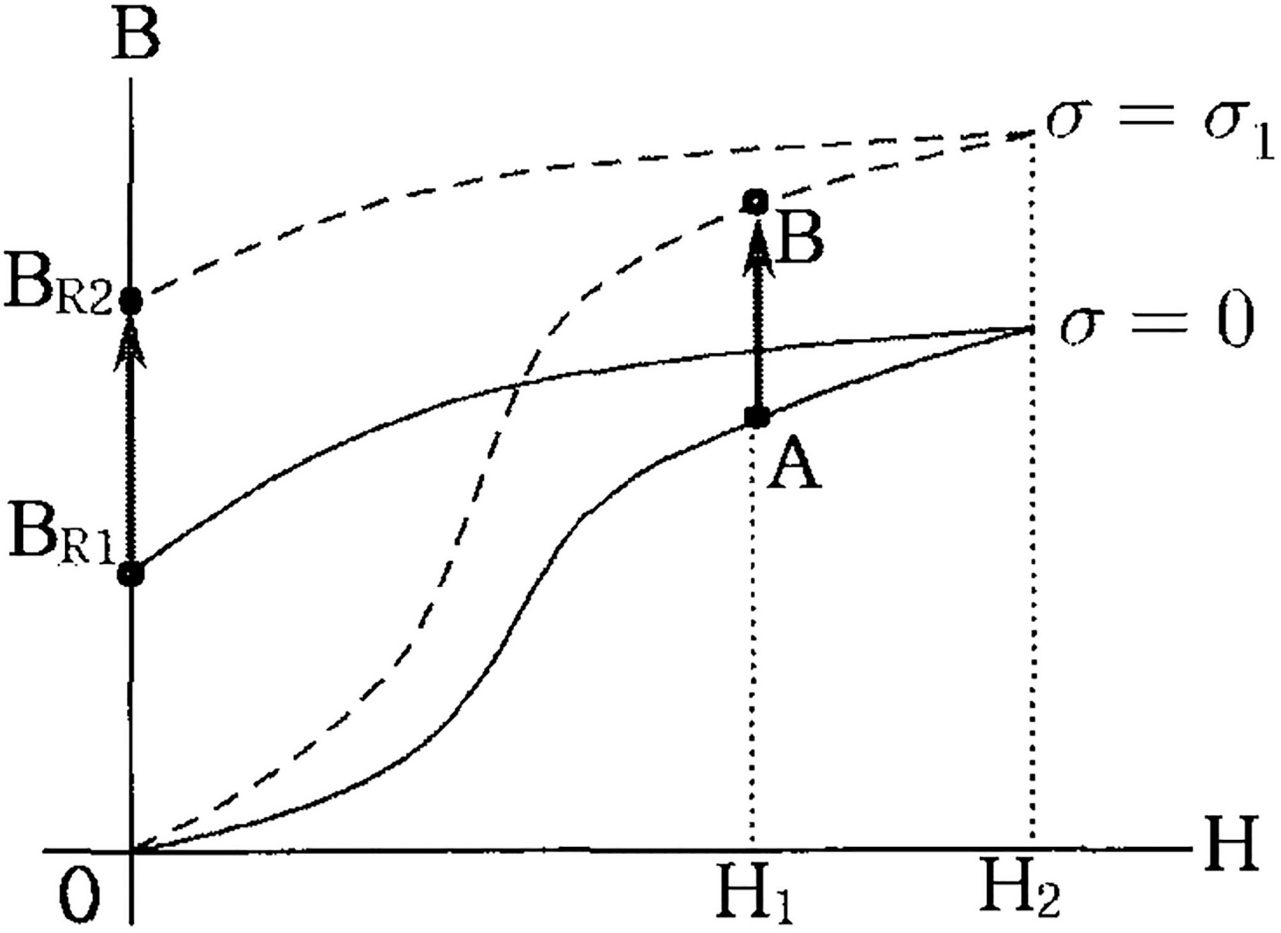
The effect of tensile force in magnetic field.

As shown in Fig 4, on the magnetic flux density–magnetic field curve (B-H curve) of the ferromagnetic material with positive magnetostrictive properties, the material is magnetized by A when the magnetic field *H*_1_ is applied in the absence of a prestress force. Similarly, the material is magnetized by B under a constant magnetic field when a prestress force *σ*_1_ is applied.

Under no prestressed force, the residual magnetic flux density is *B*_*R*1_ when the prestressed force is eliminated after increasing the magnetic field from *H*_1_ to *H*_2_. However, the residual magnetic flux density increases to *B*_*R*2_ when a prestressed force of *σ*_1_ is present.

*N*_1_: number of turns of primary coil,

*S*_1_: cross-sectional area inside the primary coil[*m*^2^],

*S*_*c*_: cross-sectional area of ferromagnetic core[*m*^2^],

*d*_1_: diameter of primary coi[m],

*l*_1_: length of primary coil[m]

*l*_*c*_: length of ferromagnetic core[m]

*N*_2_: number of turns of the secondary coil,

*S*_2_: cross-sectional area inside the secondary coil[*m*^2^],

*d*_*c*_: diameter of ferromagnetic core[m]

**Fig 5:**
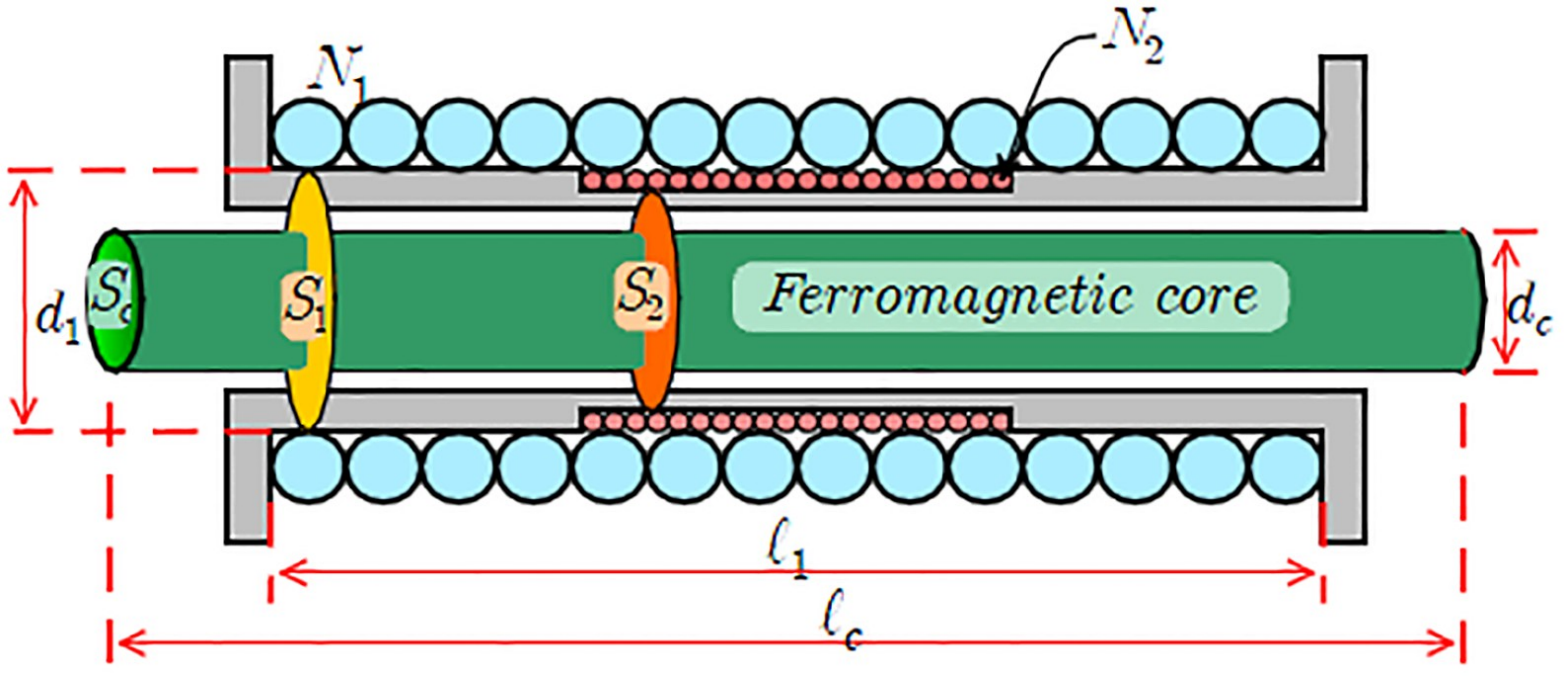
Conceptual diagram of an elasto-magnetic sensor.

However, when the magnetic field of the test specimen is eliminated, i.e., *H* = 0, the magnetization does not change even when the prestressed force is applied. The magnetic curve changes with external prestressed force because the permeability (*μ*) changes with tension. This indicates that the magnetic flux density changes with the prestressed force applied to the ferromagnetic material. Fig 5 describes the conceptual diagram of the EM sensor that can be used to estimate the tensile force of earth anchor based on this principle.

The voltage induced by the secondary coil when the current *i*_1_(t) is applied to the primary coil can be expressed using Faraday’s law, as shown in Eq (2).

$$\begin{array}{c} {\text{e}}_{2}(t)=-N_{2}\frac{d\Phi_{21}\left(t\right)}{dt} \end{array}$$ (2)

In Eq (2), Φ_21_(t) is the magnetic flux induced in the secondary coil by the primary coil and consists of the magnetic flux Φ_*c*_(t) that flows inside the magnetic material and through the air gap (*S*_*y*_ = *S*_2_ − *S*_*c*_). This can be arranged to express the magnetic flux transferred to the secondary coil, as shown in Eq (3).

$$\begin{array}{c} \Phi_{21}(t)=\Phi_{c}(t)+\Phi_{g}(t)=\kappa \;\frac{N_{1}}{l}\left(\mu S_{c}+\mu_{0}(S_{2}-S_{c})\right)i_{1}\left(t\right) \end{array}$$ (3)

where *μ* is the permeability that represents the degree of magnetization of the given magnetic field, and $\kappa =\frac{l}{\sqrt{{d_{1}}^{2}+l^{2}}}$. The voltage induced in the secondary coil can be found by substituting Eq (3) into Eq (2), as shown in Eq (4).

$$\begin{array}{c} {\text{e}}_{2}(t)=-\kappa \frac{N_{1}N_{2}}{l}\left(\mu S_{c}+\mu (S_{2}-S_{c})\right)\frac{di_{1}\left(t\right)}{dt} \end{array}$$ (4)

If permeability is known, the voltage induced in the secondary coil can be found using Eq (4). However, as permeability changes with tension, temperature, and magnetic field, the output voltage also changes. Therefore, Eq (4) can be expressed as shown in Eq (5).

$$\begin{array}{c} {\text{e}}_{2}(\sigma ,T,H,t)=-\kappa \frac{N_{1}N_{2}}{l}(\mu (\sigma ,T,H)S_{c}+\mu (S_{2}-S_{c}))\frac{di_{1}(t)}{dt} \end{array}$$ (5)

On the other hand, the induced voltage when there is no magnetic material inside the secondary coil *e*_0_(t) is expressed using Eq (6).

$$\begin{array}{c} {\text{e}}_{0}(t)=-\kappa \mu_{0}S_{2}\frac{N_{1}N_{2}}{l}\frac{di_{1}\left(t\right)}{dt} \end{array}$$ (6)

The ratio between Eqs (5) and (6) is expressed in Eq (7).

$$\begin{array}{c} \frac{{\text{e}}_{2}\left(\sigma ,\text{T},\text{H},\text{t}\right)}{{\text{e}}_{0}\left(\text{t}\right)}=\frac{\mu \left(\sigma ,\text{T},\text{H}\right){\text{S}}_{\text{c}}}{\mu_{0}{\text{S}}_{2}}+\frac{{\text{S}}_{2}-{\text{S}}_{\text{c}}}{{\text{S}}_{2}} \end{array}$$ (7)

Relative permeability (*μ*_*r*_ = *μ*/*μ*_0_), which represents the ratio between the permeability of the medium and the permeability under vacuum (*μ*_0_ = 4*π* × 10^−7^H/m), can be used to rearrange Eq (7) and obtain Eq (8).

$$\begin{array}{c} \mu_{r}(\sigma ,T,H)=1+\frac{S_{2}}{S_{c}}(\frac{e_{2}(\sigma ,T,H,t)}{e_{0}(t)}-1) \end{array}$$ (8)

Therefore, the relative permeability *μ*_*r*_(*σ*, *T*, *H*) can be obtained from Eq (8) by measuring the respective voltage induced in the presence and absence of magnetic material inside the EM sensor.

As relative permeability changes with the strength, temperature, and tensile force of the given magnetic field, the tensile force applied to the earth anchor tendons can be evaluated using the magnetic field strength and changes in relative permeability measured using the EM sensor and temperature from the thermometer. In addition to using the basic relative permeability as a parameter to estimate tensile force, the method for measuring tensile force with the width of the magnetic hysteresis curve as a parameter was used in this study to extract the change in magnetic flux density over the entire magnetic field.

## Residual tensile force measurement using magnetic properties

### Superposition of magnetic field outside the solenoid coil

**Fig 6:**
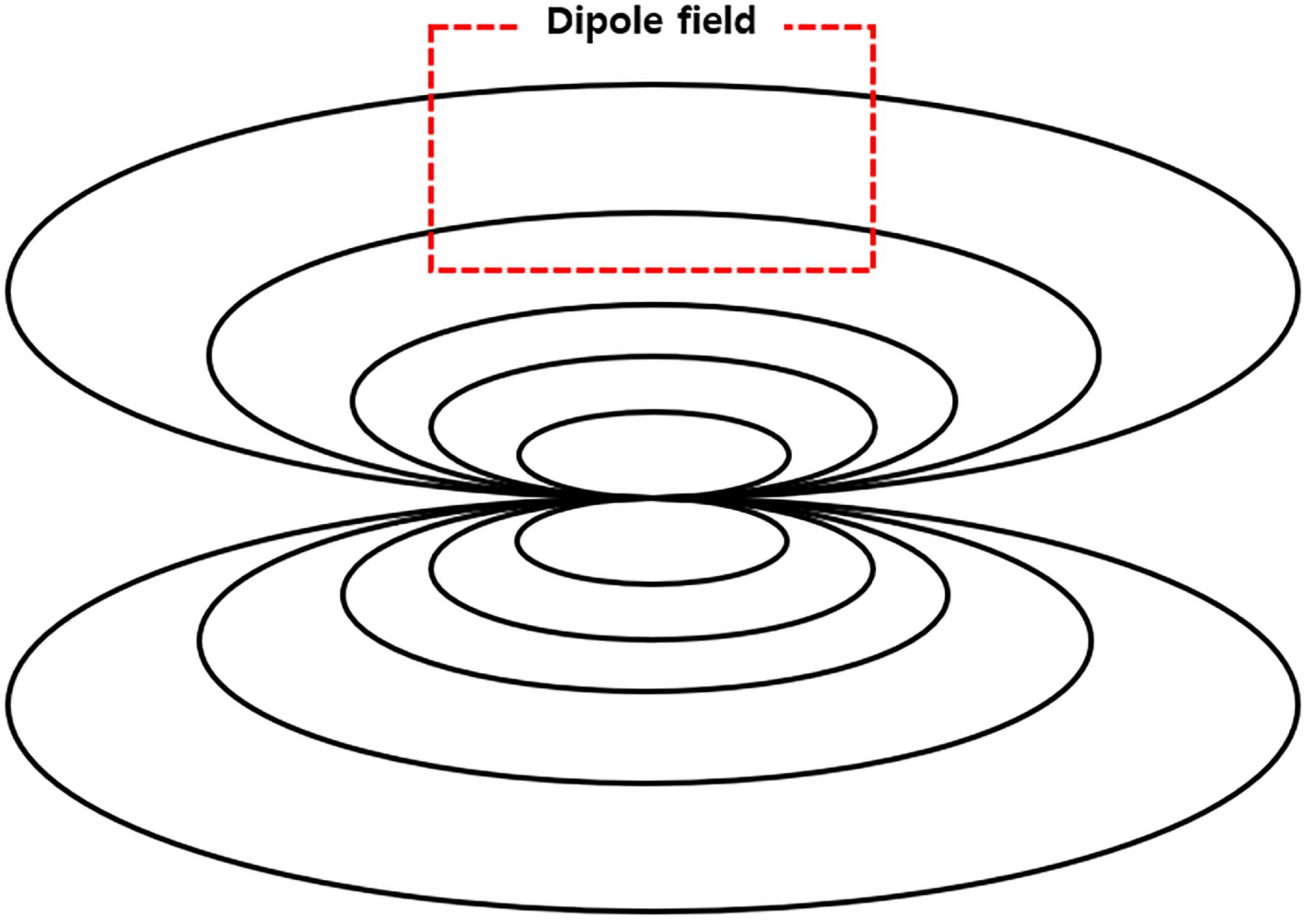
A magnetic field diagram outside a solenoid.

To understand the superposition of magnetic fields outside a solenoid coil, the size of the magnetic field generated externally by the solenoid coil needs to be identified. At a finite length, the external magnetic field of the solenoid coil is generated by the dipole moment of the solenoid, as shown in Fig 6, at a distance greater than the length of the solenoid coil.

Given the radius of the solenoid coil *R*[m], cross-sectional area *S*[*m*^2^], length *L*[m], and the number of turns per unit length *n*[turn/m], the external magnetic field of the solenoid coil can be obtained using Eq (9) or Eq (10) [22].

$$\begin{array}{c} {\text{B}}_{out}=\frac{2}{\pi }\frac{\mu_{0}nIS}{L^{2}} \end{array}$$ (9)

or,

$$\begin{array}{c} {\text{B}}_{out}=\frac{2}{\pi }\frac{\mu_{0}NIS}{L^{3}} \end{array}$$ (10)

The direction of the magnetic field in a solenoid coil is determined by Ampere’s right-hand screw rule. The magnetic fields of the same direction are compounded, while those of the opposite direction cancel each other. Therefore, it was confirmed in this study that the superposition of magnetic fields outside the solenoid coil was possible using Eqs (9) and (10), and Ampere’s right-hand screw rule. Based on this, the technique to concentrate the magnetic field around the wedge of an earth anchor was verified.

### Tensile force measurement using the area of B-H curve

The effect of stress on magnetization is generally referred to as the magneto-mechanical effect. According to the magneto-mechanical effect, stress can create an easy axis of magnetization.

$$\begin{array}{c} \begin{array}{c} {\text{E}}_{me}=-\frac{3}{2}\lambda_{100}\sigma \left(\alpha {{}_{1}}^{2}\gamma {{}_{1}}^{2}+\alpha {{}_{2}}^{2}\gamma {{}_{2}}^{2}+\alpha {{}_{3}}^{2}\gamma {{}_{3}}^{2}\right)-3\lambda_{111}\sigma \left(\alpha {}_{1}\alpha {}_{2}\gamma {}_{1}\gamma {}_{2}+\alpha {}_{2}\alpha {}_{3}\gamma {}_{2}\gamma {}_{3}+\alpha {}_{3}\alpha {}_{1}\gamma {}_{3}\gamma {}_{1}\right) \end{array} \end{array}$$ (11)

Thus, when stress is applied to the PS tendon, it causes uniaxial anisotropy, and the corresponding magneto-elastic energy *E*_*me*_ is defined as [23] where *σ* is the stress *α*_1_, *α*_2_, *α*_3_ are the direction cosines of M; and *γ*_1_, *γ*_2_, *γ*_3_ are the direction cosines of *σ* with respect to the crystal axes. The value of *E*_*me*_ changes according to the applied stress, and the residual tensile force of the earth anchor can be monitored by measuring the area of the B-H loop of the earth anchor.

## Design of EM sensor for external magnetization

### Derivation of magnetization method using numerical analysis

The design considerations of the EM sensor in this study include the number of turns of the primary and secondary coils, coil diameter, and bobbin diameter on which coils are turned. For optimal sensor design, ANSYS Maxwell, a commercial simulation program for magnetic field analysis, was used to analyze the magnetization and magnetic flux density using the EM sensor.

In particular, when an external load is applied to the magnetic material, the degree of magnetization of the material changes with the magnetic domains inside the material. The magnetic fields were simulated to confirm the degree of magnetization using the magnetic fields generated at the EM sensor. The optimal arrangement of the EM sensor coil was designed by analyzing the generated magnetic fields.

**Fig 7:**
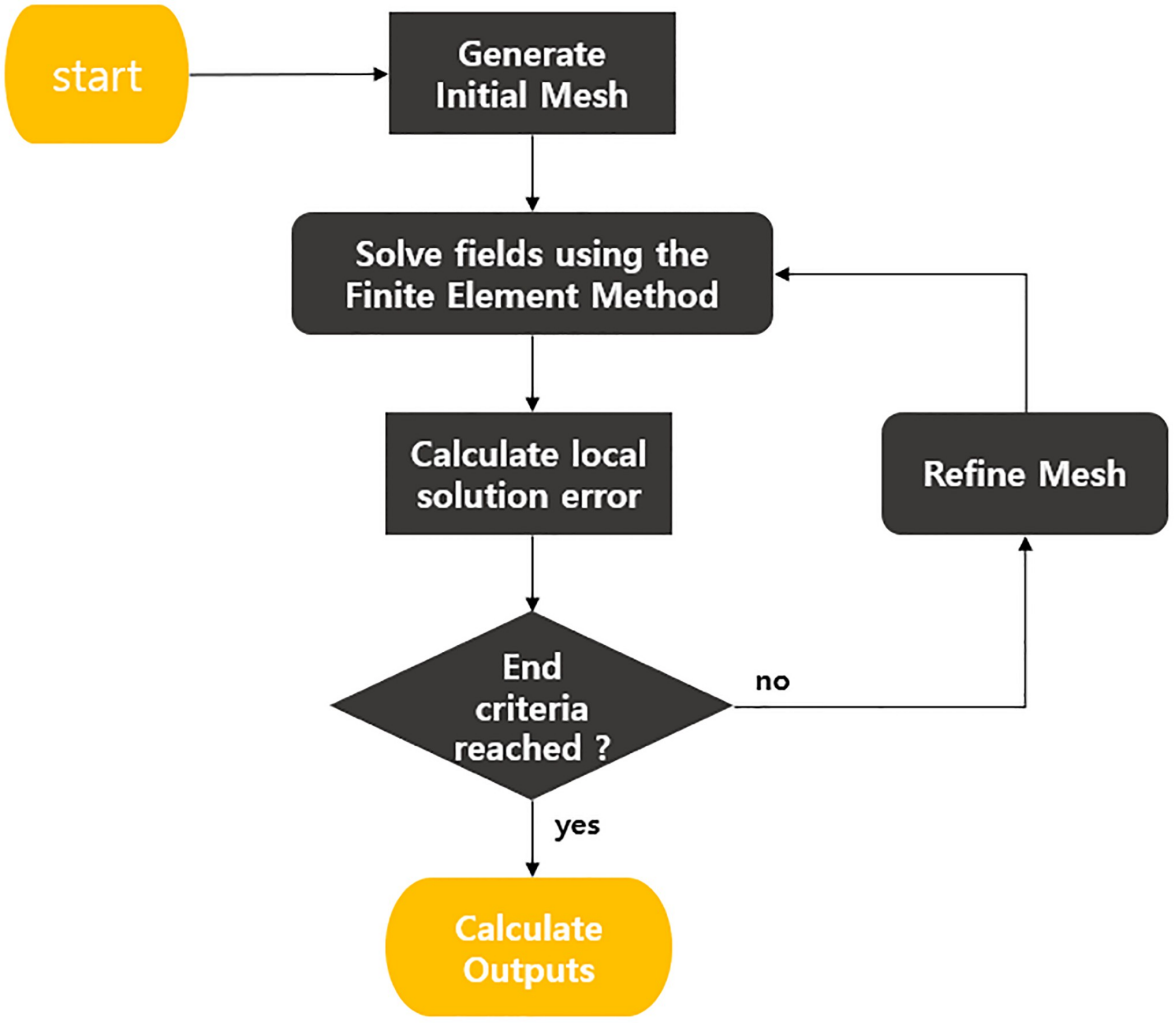
Schematic diagram of mesh setting algorithm.

Modeling of the anchorage was carried out according to the size of the actual anchorage, and in the case of the wedge of the anchorage, since it is actually divided into three divisions, modeling was carried out so that the wedge of three divisions was settled in the anchorage even in the case of modeling. In addition, the wedge is about 0.5 [mm] away from the anchorage, and through this, the mesh setting is made easier. Mesh is set to auto method, and when set to auto, Tau mesh method and classic mesh method are used at the same time. The Tau mesh method is a method that is often applied to curved surfaces, and the classic mesh method is a method that is often applied to thin or flat surfaces. In case of setting the error rate when set to Auto, the error is reduced by increasing the mesh through the algorithm as shown in Fig 7. This study tried to derive more accurate results by setting the error rate to 0.2.

For the primary coil to introduce magnetic fields, an EM sensor was modeled by turning a 60[mm] long coil wire having a diameter of 1.2[mm] in 12 layers for a total of 600[turns]. The material properties of the anchor head, wedge, tendon, and EM sensor head were set, as shown in Table 1. The magnitude of the current applied to the primary coil to generate magnetic fields was set at 15[A] considering the specification of the measurement equipment.

**Table 1:** Anchor body and sensor head properties for magnetic field analysis.

	Materials	Permittivity/Permeability	Bulk Conductivity (S *m*^−1^)	Dielectric Loss Tangent	Core Loss	Element	Mass Density (kg *m*^−3^)
*Tendon*	steel	1/B-H Curve	2,000,000	0	None	Solid	7,872
*Settlement*	steel	1/B-H Curve	2,000,000	0	None	Solid	7,872
*Acupressure plate*	steel	1/B-H Curve	2,000,000	0	None	Solid	7,872
*Wedge*	steel	1/B-H Curve	2,000,000	0	None	Solid	7,872
*EMSensor Bobbin*	polyethylene	2.25/1	0	0.001	None	Solid	930
*Coil*	copper	1/0.999991	58,000,000	0	None	Solid	8,933

**Fig 8:**
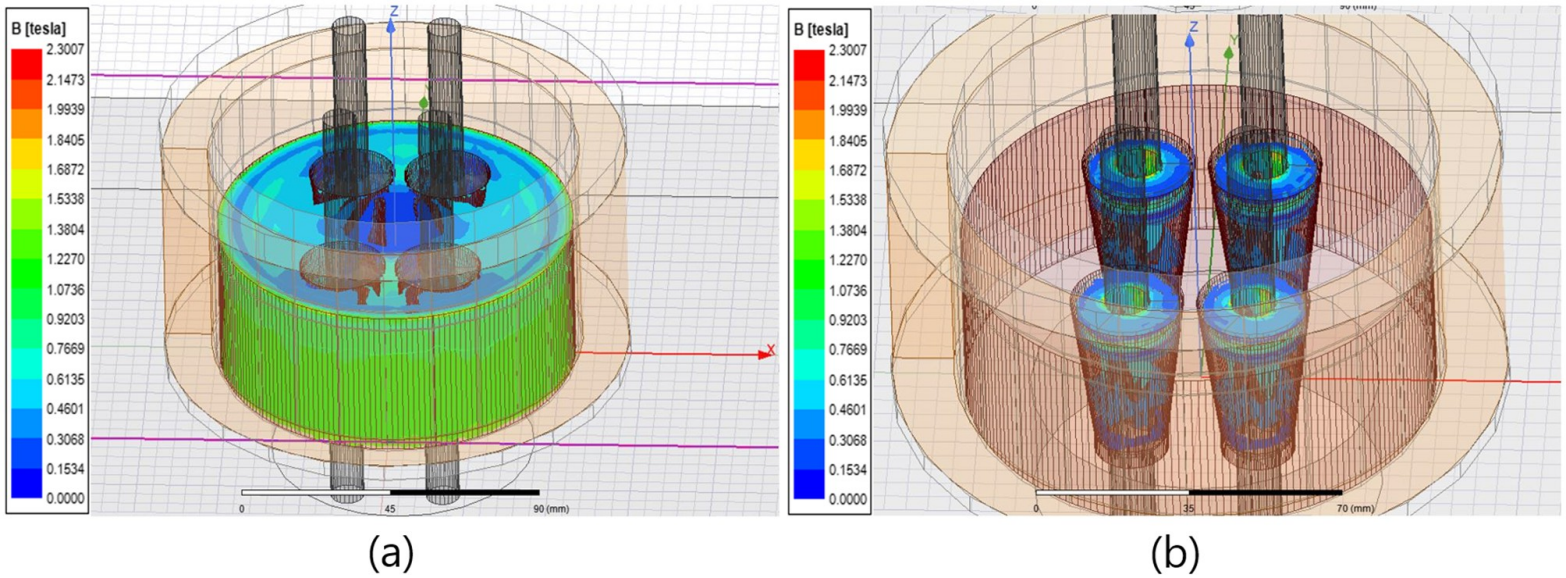
Magnetic field distribution (600[turns]) (a): Magnetic flux density distribution of the anchor head. (b): Magnetic flux density distribution of the wedge.

To successfully measure the tensile force of an earth anchor using the magnetic field, the optimal measurement position should be determined by matching the location where stress changes significantly with tensile force to the position where the magnetic field is effectively generated. The result of the magnetic field analysis indicated that it was difficult to measure using the EM sensor at the bottom of the wedge, where stress changed the most because the anchor head and bottom of the wedge were blocked by the bearing plate, causing the degree of magnetization to be underestimated. Hence, the magnetic field was observed at the anchor head and at the top of the wedge, where the load changed the most after the bottom. Additionally, it was confirmed that the magnetization of the surface of the anchor head was possible when the coil was turned higher than the top of the anchor head, as shown in the magnetic flux distribution in Fig 8(a) and 8(b).

However, the result of turning the wire 600 times indicated that the magnetic flux density was primarily generated by the anchor head and bearing plate. The magnitude of the magnetic flux density was insufficient to be measured at the wedge, which was significantly affected by the changes in tensile force. This led to an increase in the number of turns to 1000 for additional numerical analysis. Other material properties of the anchor head model and materials used in the numerical analysis were set, as described in Table 1.

**Fig 9:**
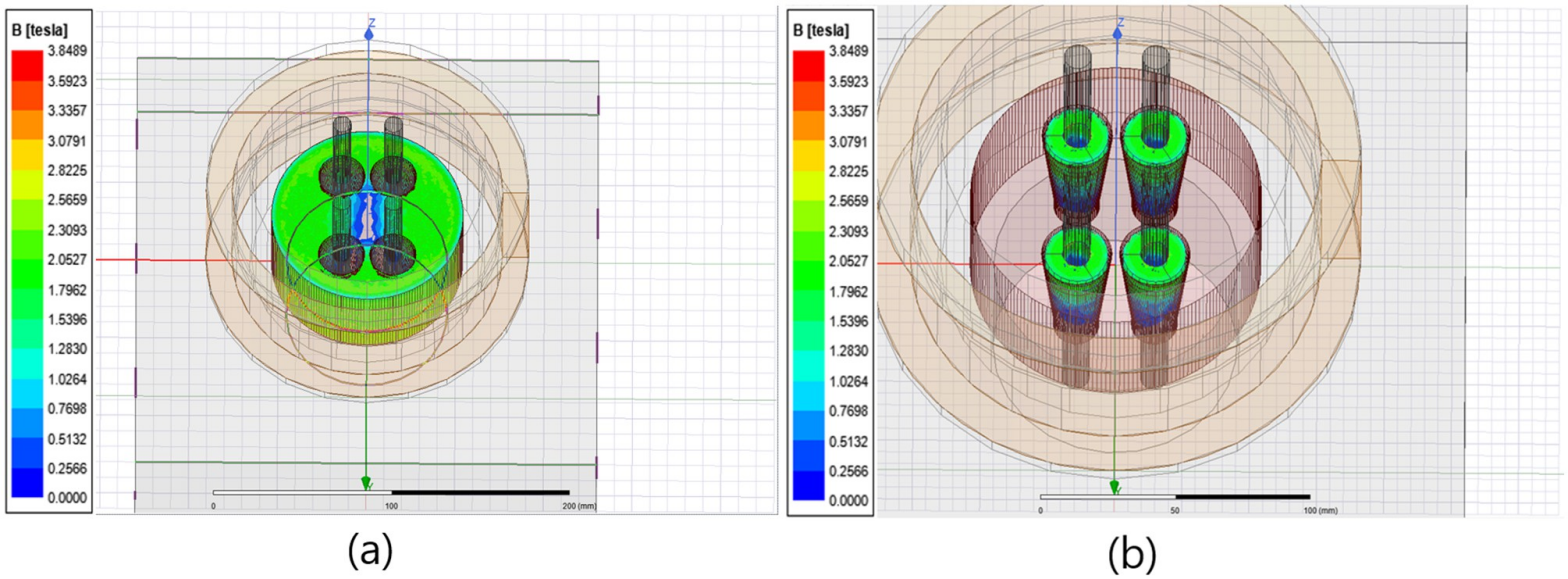
Magnetic field distribution (1000[turns]) (a) Magnetic flux density distribution of the anchor head. (b) Wedge magnetic flux density distribution.

When the number of turns of the primary coil was increased from 600 to 1000 in the conventional sensor head specification, the magnitude of the induced magnetic flux density increased from 1.2[T] to 1.9[T] on average, as shown in Fig 9, compared to the sensor with 600[turns].

However, similar to the sensor head with 600[turns], the magnetic flux density of the wedge was evaluated to be lower than 1.0[T] on average, thus confirming that increasing the number of turns in the sensor head would not increase the magnetic flux density of the wedge.

**Fig 10:**
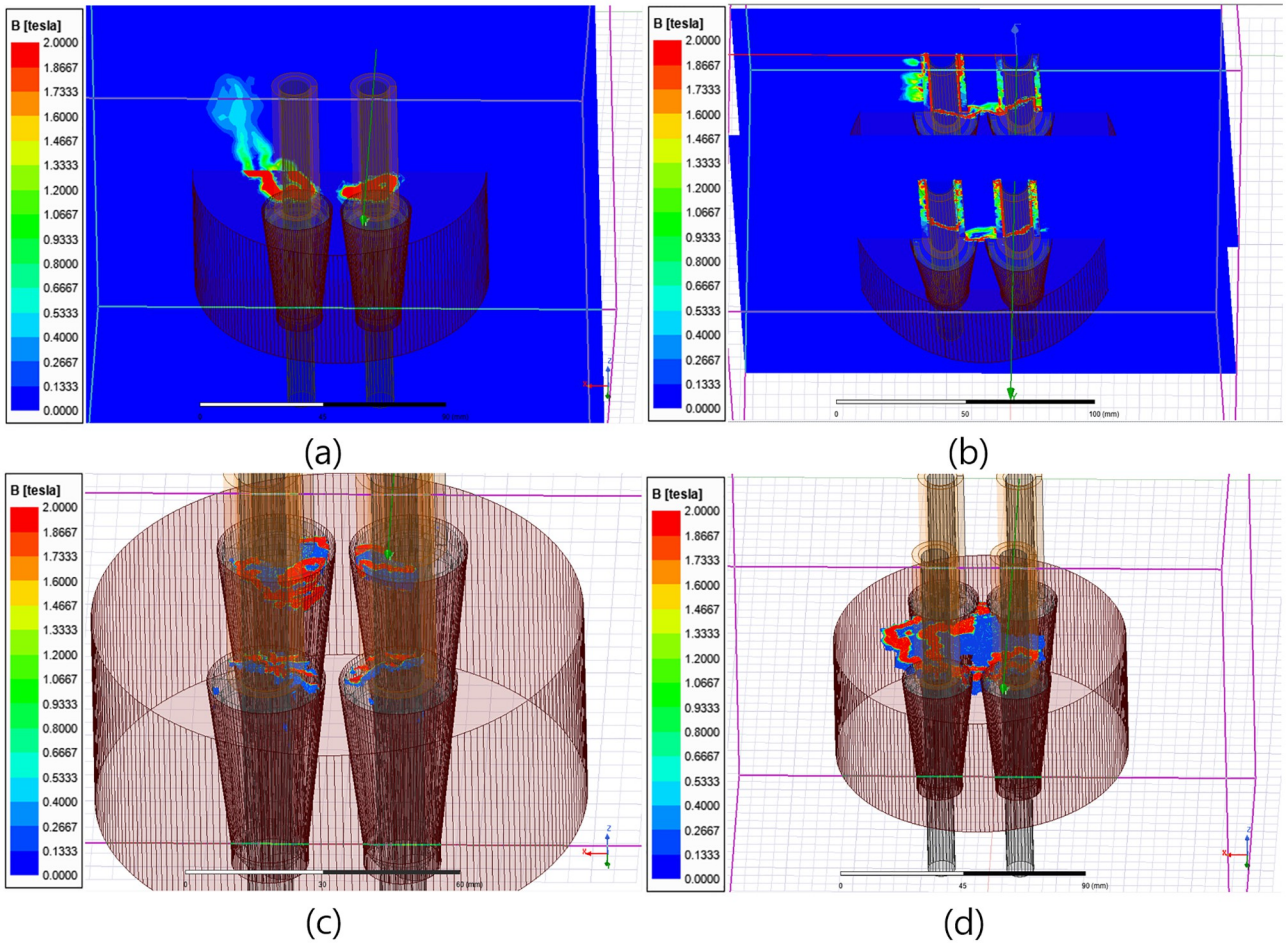
Magnetic field distribution analysis results for the cases of coil setup directly installed to individual tension member (full coil direction; counterclockwise) (a) Magnetic field distribution map (X-Z plane, whole). (b) Magnetic field distribution map (X-Z plane, per coil). (c) Wedge magnetization. (d) Anchor head magnetization.

**Fig 11:**
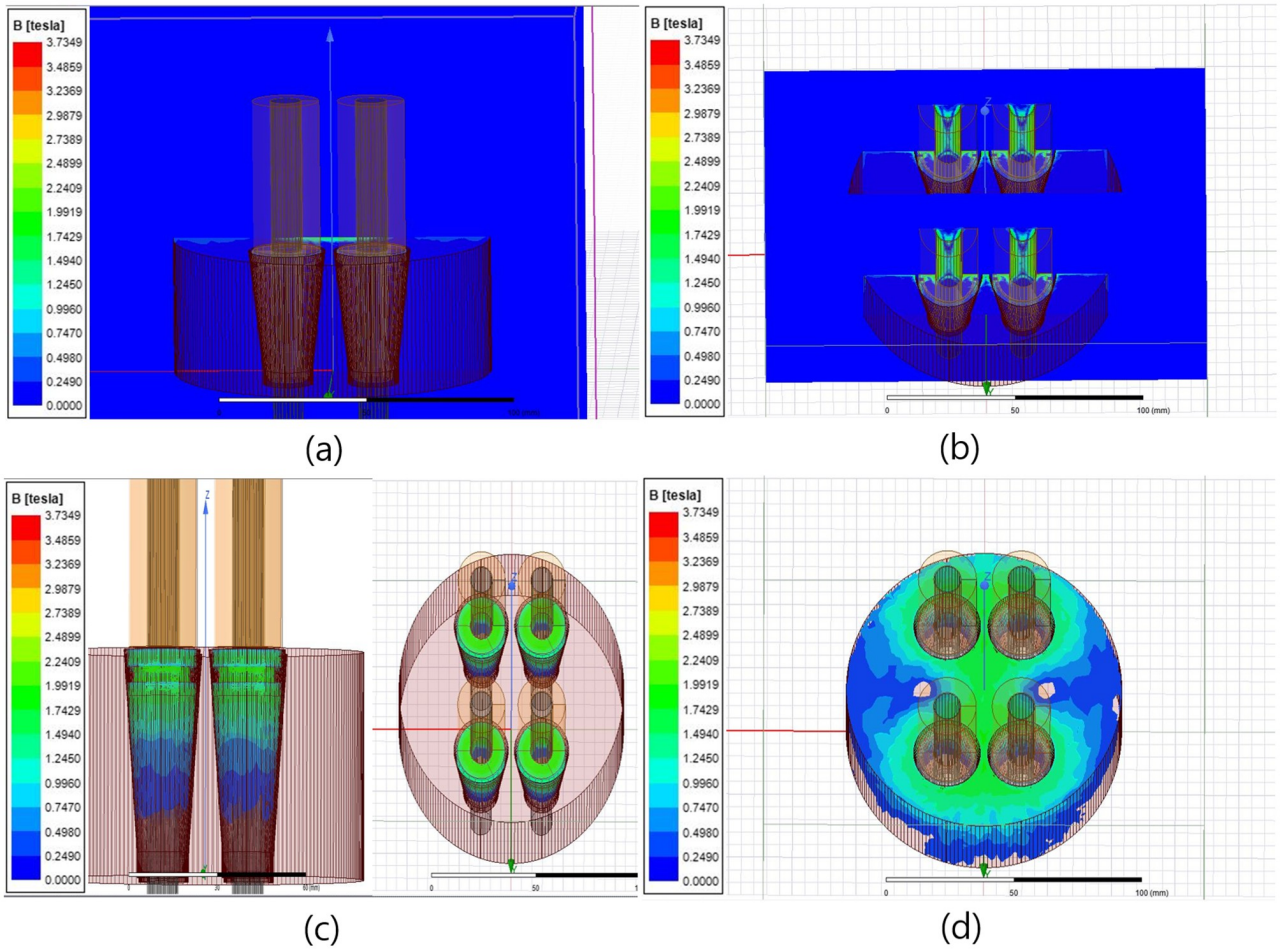
Magnetic field distribution analysis results for the cases of coil setup directly installed to individual tension member (left side coil direction; counterclockwise, right side coil direction; clockwise) (a) Magnetic field distribution map (X-Z plane, whole). (b) Magnetic field distribution map (X-Z plane, per coil). (c) Wedge magnetization. (d) Anchor head magnetization.

Therefore, the method for adding a coil to the individual tendon protruding on the wedge was considered to concentrate the magnetic field at the wedge to increase the degree of the induced magnetic field. Additionally, the magnetic flux density of the individual wedge and anchor head was analyzed using four extra coils turned 160 times at each wedge, as shown in Figs 10 and 11.

It was confirmed that the magnetic field was concentrated only at the top of the wedge when the coil direction was set counterclockwise on all four tendons, as shown in Fig 10, and the average magnetic flux density was approximately 1.5[T]. On the other hand, a magnetic flux density of 1.8[T] or higher was found at the top of the wedge and above the anchor head near the wedge when the coil direction of two tendons on the left was set to be counterclockwise and the other two on the right to be clockwise, as shown in Fig 11. Furthermore, the magnetic field was found to be generated over the entire wedge.

### Design and fabrication of EM sensor for external magnetization based on simulation results

**Fig 12:**
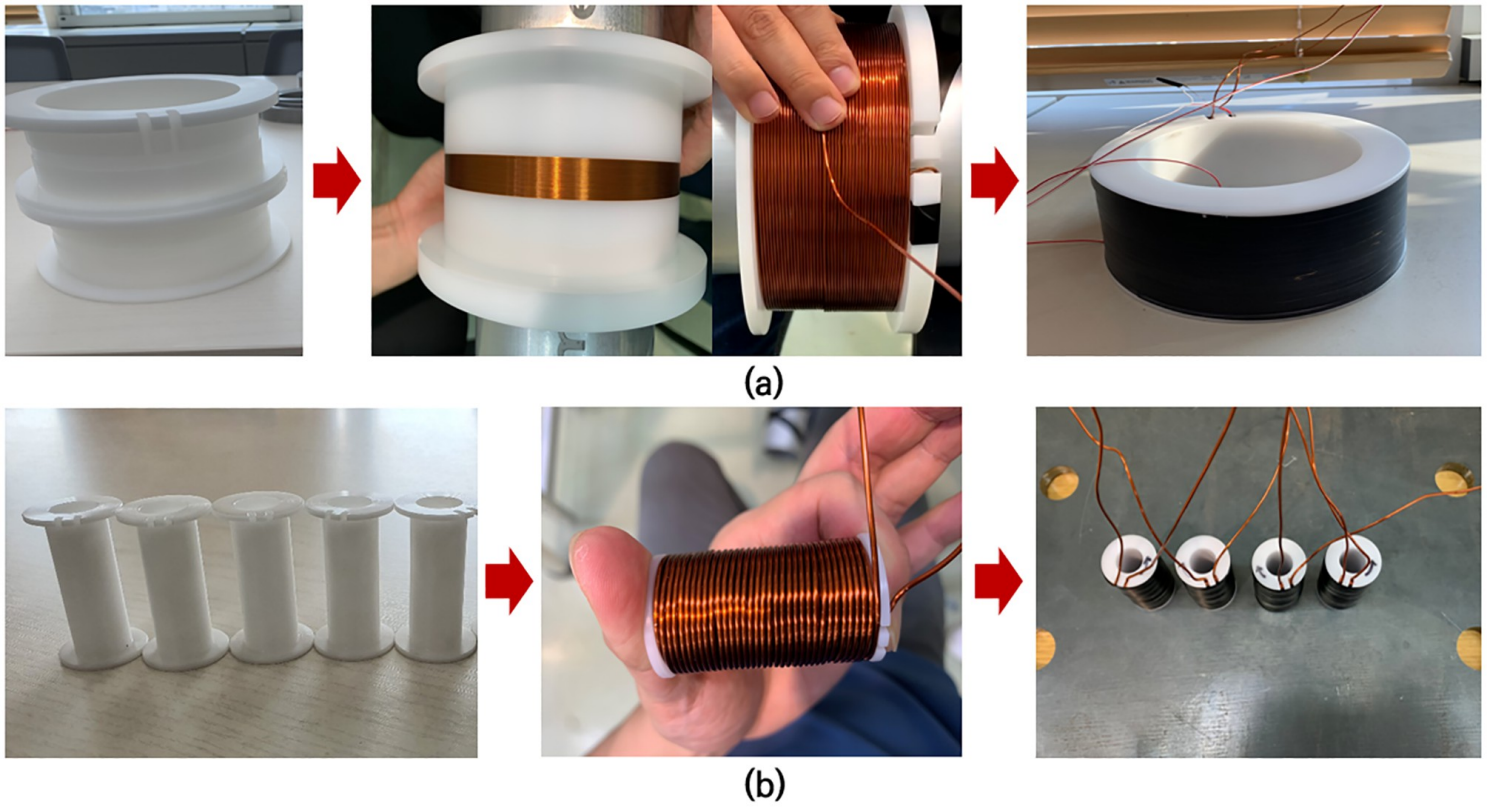
Elasto-magnetic sensor manufacturing (a) Elasto-magnetic sensor head. (b) Additional magnetization coil for the magnetization of the wedge.

Based on the numerical simulation results, the most effective method to magnetize the wedge, where stress is mostly affected by the changing tensile force of the anchor tendon, is to set the coil direction of the two left tendons counterclockwise and that on the two right tendons clockwise. Therefore, as shown in Fig 12, the coil was designed and fabricated to increase the degree of magnetization of the wedge based on the numerical simulation results.

The secondary coil wire, as shown in Fig 12(a), with a diameter of 0.3[mm] and 4[layers] was turned 160 times, whereas the primary coil wire with a diameter of 1.2[mm] and 12[layers] was turned 600 times. As shown in Fig 12(b), bobbins were designed and fabricated considering the diameter of the tendon and the number of additional coil turns. Moreover, four additional primary coils were fabricated by turning each coil 160 times.

## Experiment and results

### Experimental setup

The test to measure the tensile force of earth anchor using the EM sensor was conducted using a pedestal model for the earth anchor tensile experiment. The typical earth anchor, which had a free length of 10[m], was used for the experiment. Although it was impossible to simulate the fixed length of the anchor, the tendon was fixed using an anchor head and wedges on both ends. The tendons used in the experiment required four seven-wire steel strands with an effective cross-sectional area of 12.7[mm^2^] for each test.

**Fig 13:**
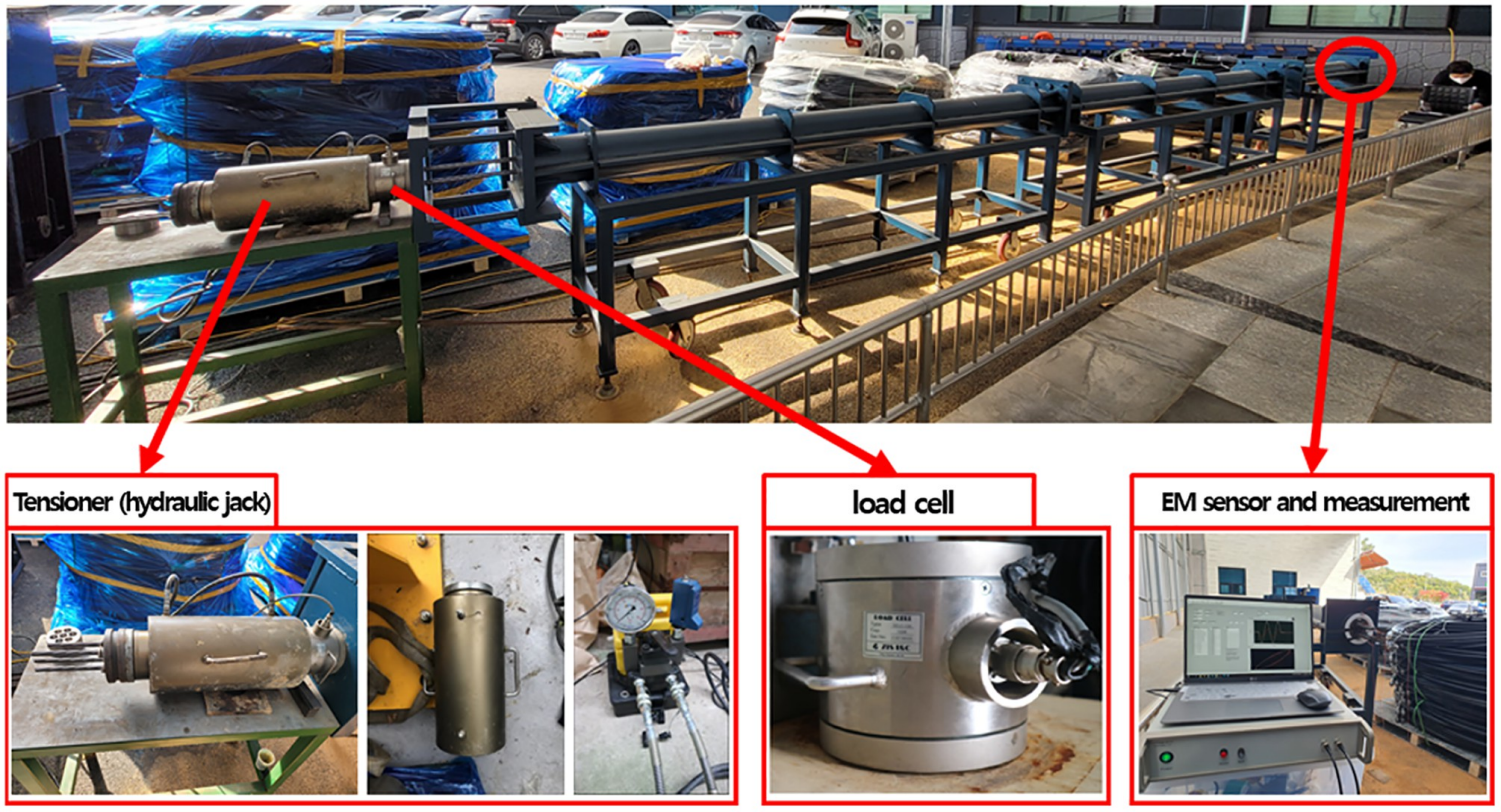
Installation of load control and measuring devices used in the test.

**Fig 14:**
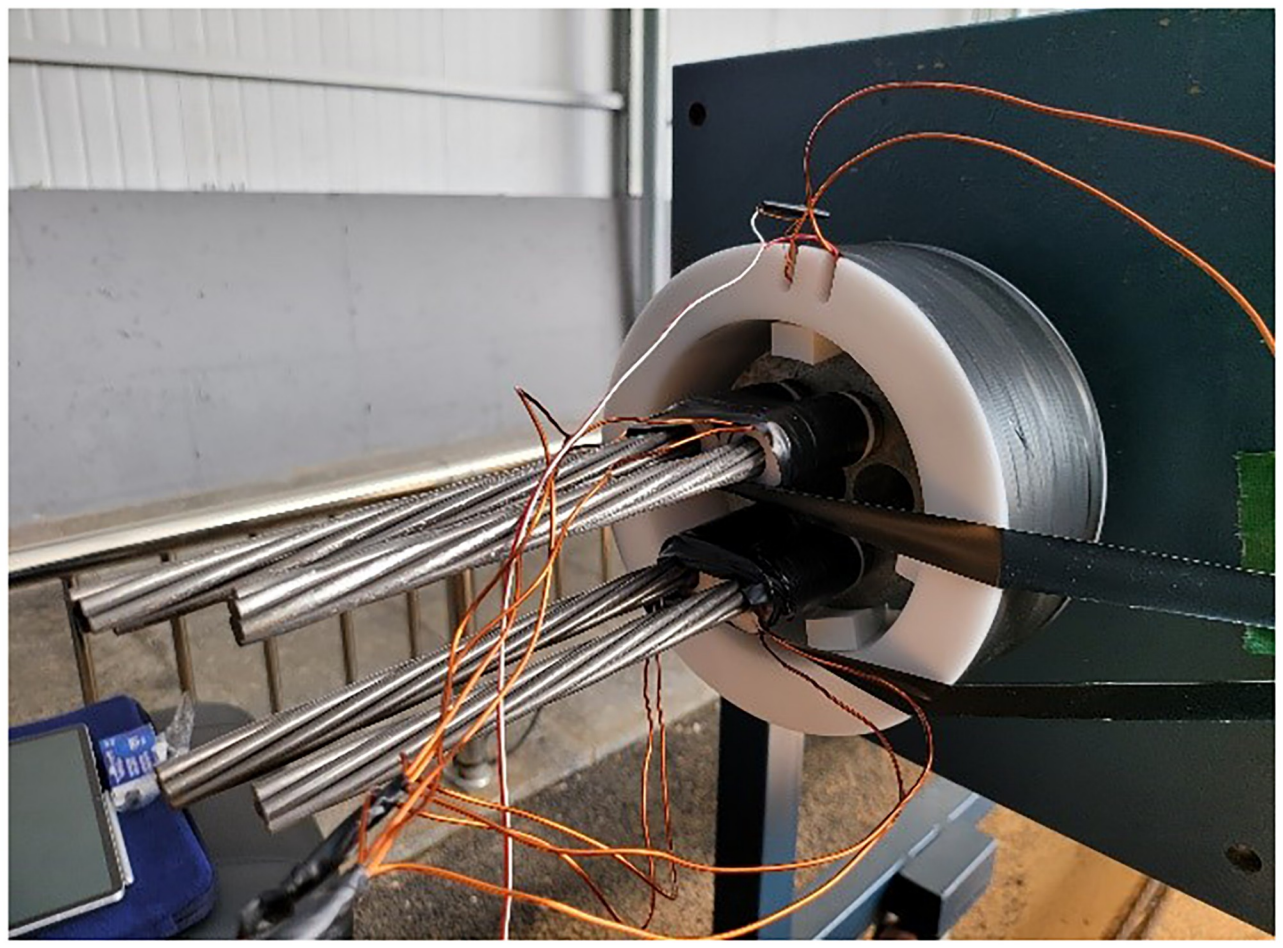
Elasto-magnetic sensor installation view.

The load control device for simulating the loss of tensile force of the anchor tendon was tested five times by installing a tensile cylinder and pump to the anchorage on the left, as shown in Fig 13. The maximum allowable tensile force of 40[tonf] was introduced to the earth anchor, which was subsequently reduced to 39[tonf]. The magnetic flux density was measured while reducing the tensile force from 39[tonf] to 5[tonf] at 2[tonf] intervals. The EM sensor was installed at anchor head, which is place opposite the side of the load control device, as shown in Fig 14.

### Magnetization measurement results

**Fig 15:**
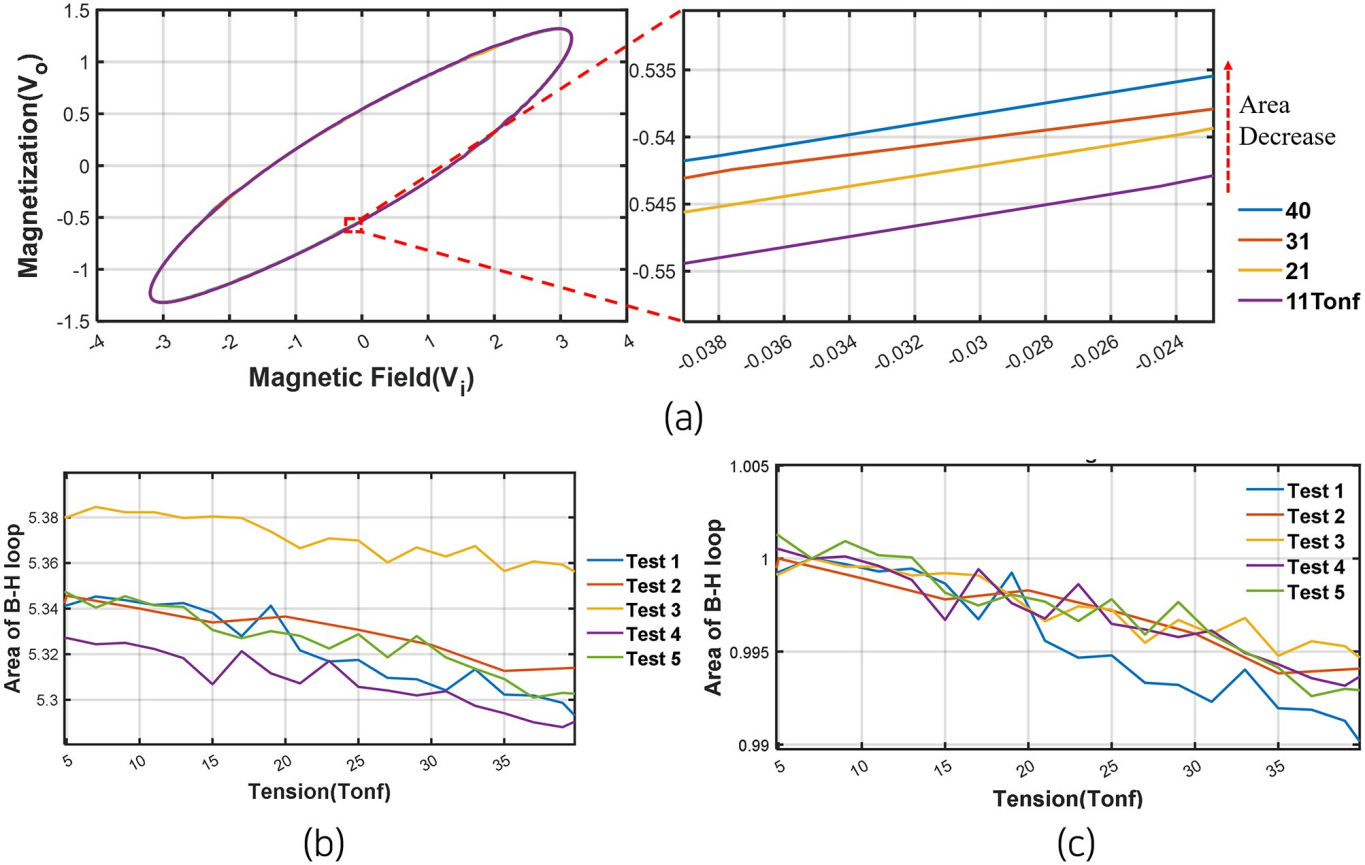
Changes in the hysteresis curve area when tension is unloaded (a) B-H curve (b) Raw area. (c) Normalized area.

As observed in Fig 15, it was confirmed that the changes in the area of B-H curve increased steadily with a decrease in residual load. It caused by the magnetic fields which induced by the primary and additional coils. Although the repeated tests resulted in a magnetic flux drift, the bias caused by the magnetic flux drift was eliminated by dividing the area at the load of 5[tonf], which was the theoretical load with the maximum area, by the overall area, as shown in Fig 15(c).

**Fig 16:**
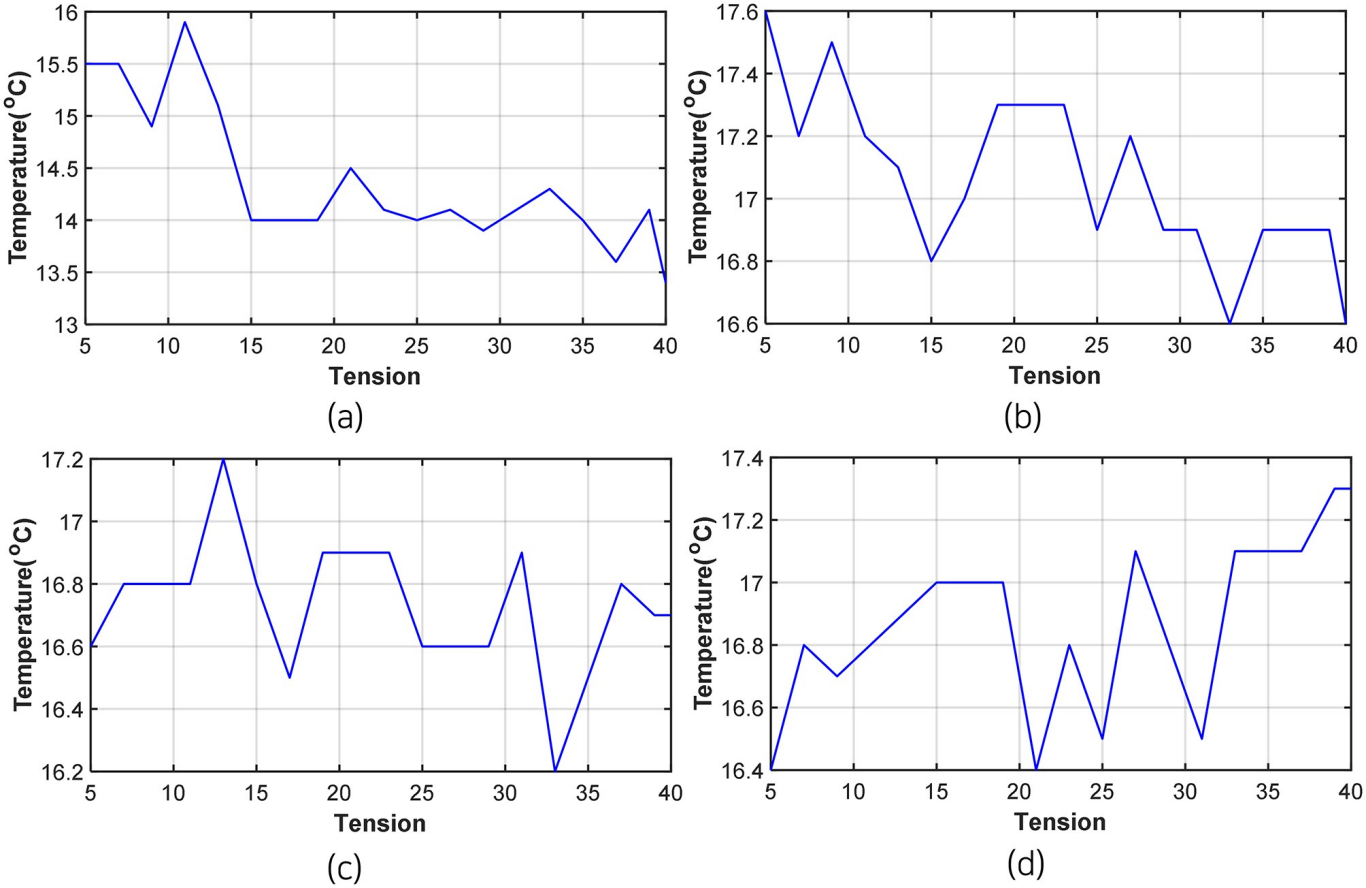
Temperature graph by test load step (a) Test 1. (b) Test 3. (c) Test 4. (d) Test 5.

However, as the area of the B-H curve changes slightly with varying tensile forces, outliers can occur owing to noise from various external environments [24]. Therefore, among the external variables, the effect of temperature was first examined in this study by measuring the surface temperature of the anchor head at each load step while measuring magnetic flux density. Table 2 and Fig 16 show the temperature distribution by load step. Test 2 was excluded because the temperature was not measured.

**Table 2:** Temperature by test load step.

	Temperature(°C)			
	Test 1	Test 3	Test 4	Test 5
40	13.4	16.6	16.7	17.3
39	14.1	16.9	16.7	17.3
37	13.6	16.9	16.8	17.1
35	14.0	16.9	16.5	17.1
33	14.3	16.6	16.2	17.1
31	14.1	16.9	16.9	16.5
29	13.9	16.9	16.6	16.8
27	14.1	17.2	16.6	17.1
25	14.0	16.9	16.6	16.5
23	14.1	17.3	16.9	16.8
21	14.5	17.3	16.9	16.4
19	14.0	17.3	16.9	17.0
17	14.0	17.0	16.5	17.0
15	14.0	16.8	16.8	17.0
13	15.1	17.1	17.2	16.9
11	15.9	17.2	16.8	16.8
9	14.9	17.5	16.8	16.7
7	15.5	17.2	16.8	16.8
5	15.5	17.6	16.6	16.4

**Fig 17:**
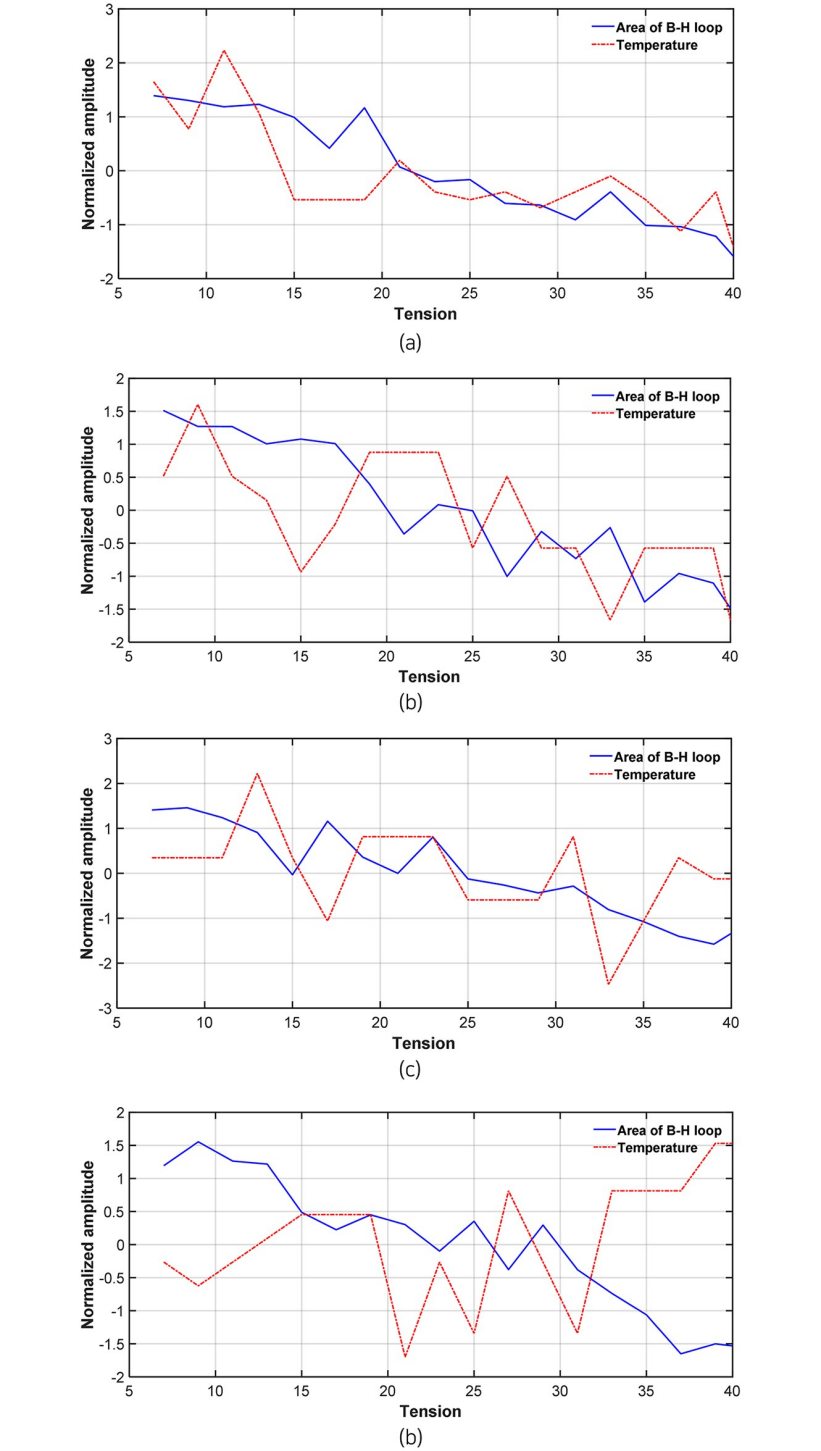
Comparison of B-H curve area and temperature change by test load step (amplitude is normalized) (a) Test 1. (b) Test 3. (c) Test 4. (d) Test 5.

To correlate the load and temperature measured in each test, the temperature data and B-H curve area were normalized to adjust the amplitude size, as shown in Fig 17. The correlation coefficient between the B-H curve area and the temperature distribution was calculated using the normalized data, as provided in Table 3. The correlation coefficient in Test 1 was found to be the largest, but the value was only approximately 0.7. Additionally, a negative correlation was seen in Test 5. Therefore, the effect of temperature was considered to be insignificant.

**Table 3:** The correlation coefficient between area and the temperature of B-H curve.

	Test No.			
	1	3	4	5
Correlation Coefficient	0.7209	0.4870	0.3706	-0.5808

**Fig 18:**
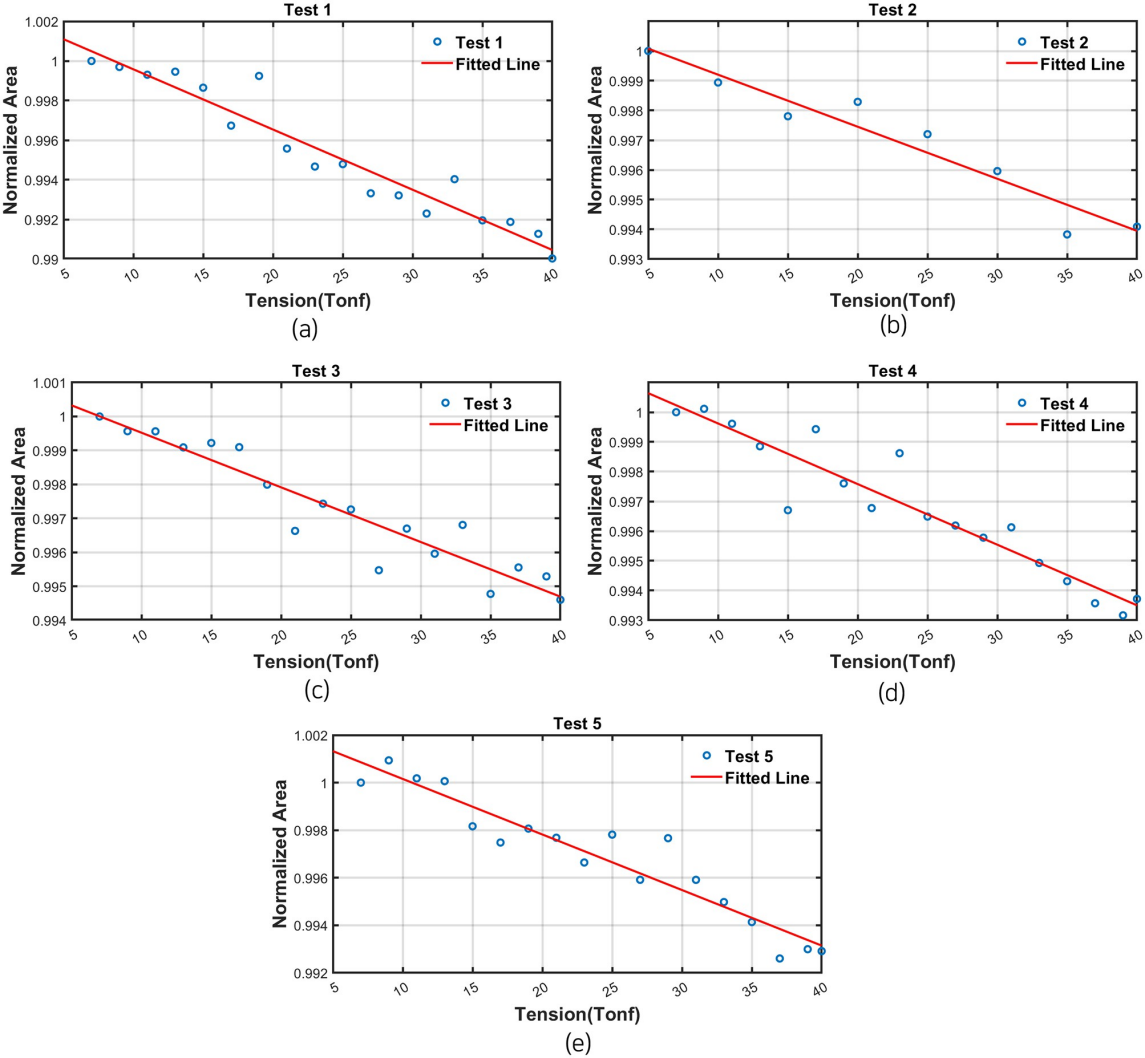
Linear regression results (a) Test 1. (b) Test 2. (c) Test 3. (d) Test 4. (e) Test 5.

To correlate the area value of the B-H curve to the load step value of the entire derived data, linear regression was performed. The results of linear regression of each test was shown in Fig 18 and Table 4. The confidence interval was set to be 95% with the x-axis variable as tension and the y-axis variable as the B-H curve area. As shown in results, the R-square value was almost 0.9 in all tests and it means the relationship between the area of B-H curve and residual tension has linear relationship. From this result, the residual tensile force of earth anchor can estimate using the area of B-H curve measured by external EM sensor.

**Table 4:** The correlation coefficient between area and the temperature of B-H curve.

	Test No.				
	1	2	3	4	5
Slope	-3.041E-4	-1.751E-4	-1.609E-4	-2.039E-4	-2.336E-4
R^2^	0.9262	0.9274	0.8958	0.8904	0.9049

### Tensile force estimation and error analysis

**Fig 19:**
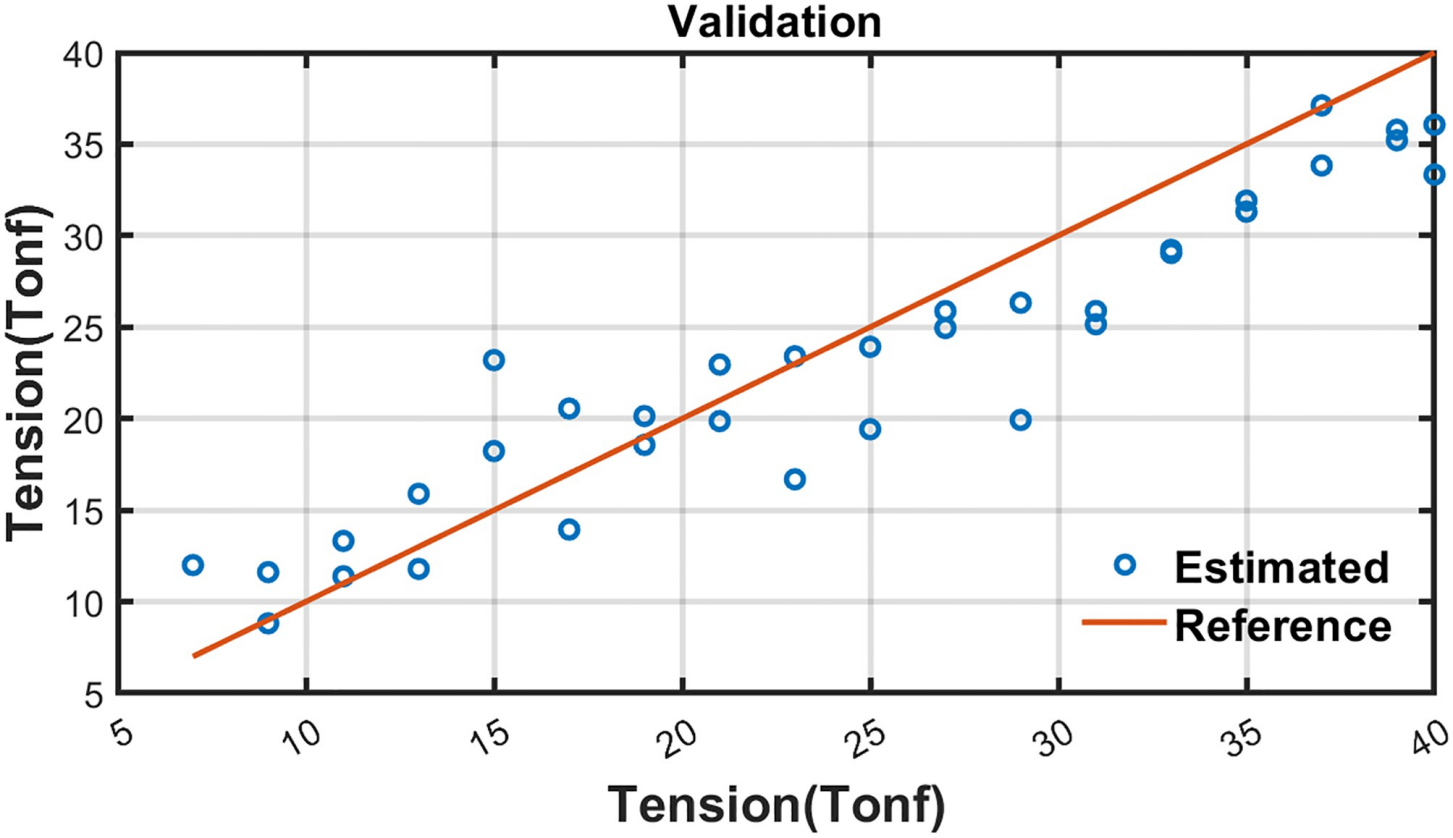
Validation results of residual tensile force estimation.

It is confirmed that the residual tensile force of earth anchor has linear relationship with normalized area of B-H loop which measured by proposed EM sensor. To estimate the residual tensile force of earth anchor, the tensile force estimation equation was derived using the data of test 1 to 3 as below.

$$\begin{array}{c} {\text{E}}_{r\_est}\left(Tonf\right)=-3396A_{n}+3408 \end{array}$$ (12)

where *T*_*r*_*est*_ is the estimated tensile force and *A*_*n*_ is normalized area of B-H loop. The proposed residual tensile force estimation method was validated using the data from Test 4 and 5 which were not used to derive the equation. Fig 19 and Table 5 shows the validation results of residual tensile force estimation.

**Table 5:** Validation results of tensile force estimation for Test 4 and 5.

Ref. Ten	Tonf			
	Test 4		Test 5	
	Est. Ten	error(%)	Est. Ten	error(%)
7	11.99939	4.999	11.99728	4.997
9	11.61299	2.613	8.817337	-0.183
11	13.31996	2.320	11.38709	0.387
13	15.89514	2.895	11.78846	-1.212
15	23.19834	8.198	18.2302	3.230
17	13.94112	-3.059	20.55218	3.552
19	20.14252	1.143	18.56538	-0.435
21	22.95594	1.956	19.86745	-1.133
23	16.68651	-6.313	23.40499	0.405
25	23.92495	-1.075	19.42396	-5.576
27	24.95109	-2.049	25.87687	-1.123
29	26.33346	-2.667	19.93241	-9.068
31	25.15675	-5.843	25.88421	-5.116
33	29.22312	-3.777	29.05475	-3.945
35	31.31023	-3.690	31.91231	-3.088
37	33.83146	-3.169	37.11522	0.115
39	35.20765	-3.792	35.78786	-3.212
40	33.33629	-6.664	36.0626	-3.937

The mean error of estimated residual tensile force was 3.248 Tonf while in two cases, a large error appeared (9.068, 8.198). The error can be reduced according to the further studies to improve input voltage and sensor.

According to the estimation results, it is confirmed that the proposed method can be one of solution for residual tensile force estimation of installed earth anchor without lifting of tendons.

## Conclusion

As a preliminary study on the measurement of residual tensile force of earth anchor, a magnetic field-based EM sensor was designed and fabricated in this study to conduct tests for measuring the tensile force of earth anchors. Tendon, which is a type of cable structure that is loaded with tension, is the most important structural member within earth anchors. As cable structures may lose some design tension or tensile force during construction owing to various factors, it is crucial to monitor the loss of the introduced tension to ensure structural stability and safety while the structures are being used. In the conventional method for measuring the tension of cable structures using magnetic field sensors, the magnetic field sensors were installed on the cables to directly magnetize the cables. The magnetic flux density was measured as a magnetic response of the cables. The variation in magnetic flux density was analyzed to estimate the tensile force of the cables. However, earth anchor tendons were buried in sloped grounds, making it impossible to install magnetic field sensors directly on the tendons of existing earth anchors as in the conventional method. Therefore, this study aimed to develop an EM sensor that could be installed on an externally exposed anchor head rather than directly on the tendon. The changes in stress and magnetic field distribution of the anchor head with tensile force were analyzed through numerical analysis. Based on this, tension measurement tests were conducted using the earth anchor pedestal model that simulated field conditions. The conclusions drawn from this study are as follows:

1. The numerical simulation to design and manufacture EM sensors confirmed that the changes in the magnetic field were concentrated at the top of the wedge and anchor head (externally exposed area). However, closed magnetic field curves could not be generated owing to the steel bearing plate, while the leakage of the magnetic field through the bearing plate was observed. Therefore, it was necessary to identify a method to magnetize the wedge that was directly affected by the changing tensile force.
2. It was confirmed through numerical simulation that the magnetic flux density could be increased by increasing the degree of magnetization of the wedge, where stress changed with tensile force, by installing additional coils to the externally exposed tendon at the anchor head and directly magnetizing the wedge. Based on the numerical simulation results, additional primary coils were designed and manufactured. Furthermore, experiments were conducted by installing these primary coils. In total, five tests were conducted that demonstrated a constant pattern of changes in the magnetic flux density under load. However, as the magnitude of the tension introduced to the earth anchor was not large, the variation in magnetic flux density with tensile force was small. Additionally, the outliers caused by noise and the influence of the external environment were also measured.
3. To analyze the effect of temperature as an external influence, the surface temperature of the anchor head was measured for each test and load step. The correlation between the magnetic flux density and temperature indicated that the effect of temperature on the magnetic flux density was insignificant.
4. To estimate the residual tensile force of earth anchors, the estimation equation was derived using test data set and it was verified using validation data set. The average error is 3.248, and it is verified that the residual tensile force can be estimated using the proposed method.

The experimental results of this study confirmed that the magnetic flux density measured as a magnetic response at the anchor head varied constantly with the changing tensile force of the earth anchor when the effect of temperature was insignificant. However, the measurement of magnetic flux density revealed magnetic flux leakage occurring at the anchor head and bearing plate. Therefore, it is necessary to increase the degree of magnetization and identify additional measures to eliminate outliers in future studies.
